# In Situ Infrared and Raman Analysis of Structural Changes of (C_60_Pd_3_)*_n_* Coordination Polymer under Elevated Pressure

**DOI:** 10.1021/acs.jpcb.5c08143

**Published:** 2026-04-14

**Authors:** Sylwia Zięba, Emilia Grądzka, Adam Mizera, Dariusz Pogocki, Barbara Seroka, Jakub Goclon, Ryszard Łaźny, Krzysztof Winkler

**Affiliations:** † Institute of Molecular Physics, 119441 Polish Academy of Sciences, M. Smoluchowskiego 17, 60-179 Poznań, Poland; ‡ Department of Chemistry, 49663 University of Bialystok, K. Ciołkowskiego 1K, 15-245 Bialystok, Poland; § 86904 Institute of Nuclear Chemistry and Technology, Dorodna 16, 03-195 Warsaw, Poland

## Abstract

Structural changes of a fullerene–palladium (C_60_Pd_3_) coordination polymer under increased pressure conditions were monitored via in situ infrared and Raman spectroscopy. The body-centered cubic structure of the fullerene-based coordination polymer could be transferred to the amorphous (C_60_)*_n_* homofullerene polymeric phase under a mild pressure of ∼4.5 GPa. This phase was formed by C_60_Pd_3_ under considerably milder pressure conditions than C_60_ polymerization that required higher pressure. Analogous investigations performed with C_60_[η^2^-Pd­(PPh_3_)_2_] complex did not form the (C_60_)*_n_* homofullerene polymeric phase. The transformation of C_60_Pd_3_ to an amorphous homofullerene polymer phase was possibly because of the formation of metallic palladium nanocrystals and a local pressure increase, resulting in the formation of (C_60_)*_n_*. Additional theoretical calculations showed that pressure strongly influenced the bonding of fullerene moieties in the presence of palladium clusters. This effect was particularly significant in the case of C_60_ moiety linked through palladium atoms.

## Graphical abstract

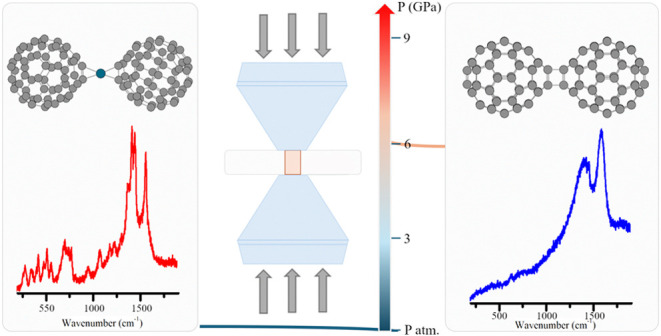

## Introduction

Fullerenes exhibit hollow cage-like structures comprising carbon atoms exclusively, which are arranged in patterns of hexagons and pentagons. C_60_, the most common fullerene, comprises 12 pentagons separated by 20 hexagons. They are connected through C–C bonds between hexagons and pentagons and poorly delocalized C=C bonds between hexagonal rings. Therefore, C_60_ behaves like an electron-deficient alkene. Such unique three-dimensional (3D) spheres containing 30 relatively reactive double bonds make fullerene C_60_ cages highly susceptible to inorganic and organic surface modifications and provide possibilities for creating various macromolecular structures.^1–5^ Fullerene-based polymers have been particularly investigated.^4–8^ Depending on the position of the fullerene unit within the polymer chain, different classes of C_60_-based polymers can be prepared.

Fullerene moieties can be covalently incorporated into a polymeric chain or backbone. A large group of fullerene-based materials comprises organic conducting polymers, with fullerenes attached covalently to the polymer chain via a linker.^9–12^ The physicochemical properties of these materials depend on the polymer chain, linker structures, and the density of fullerene moieties within the polymeric material. The organic polymer backbone exhibits electronic conductivity in out-of-chain fullerene-containing polymers. Thus, charge transfer is possible through the polymer chain and fullerene moieties attached to the main backbone as side chains.^13,14^ Such double cable structures exhibit conductivity in two different potential windows.

Fullerene moieties in in-chain structures are separated by short organic conjugated linkers,^15–18^ small inorganic linkers,^18–20^ or metal atoms and metal complexes.^6,21–27^ They can also form homopolymeric structures through [2 + 2] cycloaddition.^28–32^ This reaction is induced by excitation with photons,^28,33–35^ electrons,^29,36^ plasma discharge,^37^ or the application of a high hydrostatic pressure.^29,36^ Fullerene homopolymers were formed during the reaction of alkali metal with fullerenes,^32,38–41^ which exhibit orthorhombic (*Immm*), tetragonal (*P*4_2_/*mmc*), and rhombohedral (*R*3*m*) phases.^42^

Fullerene moieties are connected through metallic units in metallofullerene polymers such as palladium [Pd(0)] or platinum [Pt(0)] atoms, bis­(trimethylphosphine)­nickel(0) {Ni(0)­[P­(CH_3_)_3_]_2_}, bis­(carbonyl)­iridium dimer [Ir­(I)­(CO)_2_], and ruthenium­(I) triflate [Ru­(I)­(OOCCF_3_)] moieties to form a polymeric network. The chain structure of the coordination fullerene polymer comprises C_60_ moieties and palladium atoms (Scheme 1). These coordination polymers can be formed chemically in solution containing C_60_ or C_70_ and metalloorganic precursors. They can also be formed during electrochemical reduction in solutions containing fullerene and the appropriate transition metal complex. Depending on the molar ratio of the precursors for polymerization, one-, two-, or 3D polymeric structures are produced. Benzene solution containing Pd(0) complexes and C_60_ with a molar ratio close to 3:1 forms a 3D C_60_Pd_3_ structure, exhibiting an ordered body-centered cubic structure of fullerene units with a slight rhombohedral distortion.^43^ The fullerene cage is octahedrally coordinated by six Pd atoms. Each Pd atom is bonded to two C_60_ units. These coordinated polymers are electrochemically active in the negative potential range because of fullerene cage reduction. They also exhibit conducting properties in their reduced state.

**Scheme 1:**
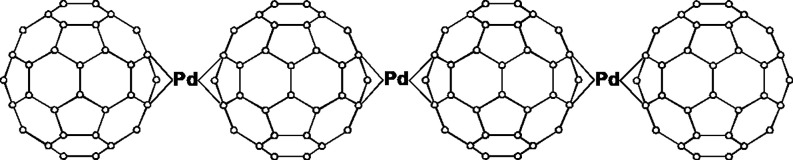
1D Chain Structure of C_60_Pd Fullerene Coordination Polymer

Fourier-transform infrared (FTIR) and Raman spectroscopy are widely used to analyze the structures of conducting polymers such as conformations, configurations, conjugation length, and degree of defects.^31,35,42,44^ These techniques can also be used for investigating the doping processes of conducting polymers. Polarons and bipolarons formed during doping can be identified via Raman spectroscopy. IR spectroscopy is a powerful tool for detecting the photogenerated and injected carriers in conducting polymers because the charge carriers increase IR band intensities.

Herein, an in situ study on the structural changes of C_60_Pd_3_ coordination polymer under increasing pressure conditions is reported. A special cell for in situ study of polymer vibration spectra during pressure changes was designed. With increasing pressure, the C_60_Pd_3_ polymer decomposed to form planar C_60_Pd_2_ polymeric network, which then participated in the formation of the C_60_ homopolymeric phase. The pressure required to form this phase was considerably lower than that required for the formation of (C_60_)*_n_* from pristine fullerene.

## Methods

Benzene (99.8%) for the synthesis of the C_60_Pd_3_ polymer was used as procured from Sigma-Aldrich. Fullerene C_60_ (99%) and tris­(dibenzylideneacetone) dipalladium(0)-chloroform adduct, Pd_2_(dba)_3_·CHCl_3_, used for coordination fullerene polymer synthesis, were purchased from Aldrich Chemical Co. and used without additional purification. Toluene and hexane used for the synthesis of the C_60_[η^2^-Pd­(PPh_3_)_2_] complex were purchased from Avantor and distilled under Ar with Na/benzophenone before usage. Pd­(PPh_3_)_4_ (99%, Sigma-Aldrich) was used without additional purification.

C_60_Pd_3_ was chemically polymerized in benzene solution containing C_60_ and Pd_2_(dba)_3_·CHCl_3_ as the polymerization precursors using a molar ratio of Pd/C_60_ of 1:3. The polymerization process was performed for 24 h at 20 °C under an argon atmosphere. The resulting C_60_Pd_3_ deposit was washed several times with pure benzene and dried at 60 °C. The composition and structure of the formed polymer were confirmed via X-ray diffraction (XRD) and thermogravimetric studies. Powder diffraction studies were carried out using a Supernova diffractometer (Agilent Technologies) operating at 50 kV and 0.8 mA and equipped with a CCD detector and a source: Cu Kα 1 (λ = 1.54056 Å). The distance between the sample and detector was 158 mm. The X-ray powder diffraction (XRD) pattern is presented in Figure S1 in the Supporting Information. The obtained XRD pattern exhibits signals typical for C_60_Pd polymeric materials in the range between 10 and 38 2Theta, which was reported by Talyzin et al.^45^ Low intensity signals of palladium nanoparticles are also observed. Formation of a small amount of palladium nanoparticles was reported in the literature.^45,46^

C_60_[η^2^-Pd­(PPh_3_)_2_] complex was synthesized according to the procedure described by Liu et al.^47^ To the solution of C_60_ (120 mg; 0.166 mmol) in toluene (20 mL), Pd­(PPh_3_)_4_ (196 mg, 0.17 mmol) was added. The solution color changed to black–green immediately. The reaction was performed in a Schlenk tube for 4 h at room temperature under an argon atmosphere. The mixture was transferred to a Falcon tube, and the precipitate was centrifuged, washed twice with 40 mL of hexane, and dried in an argon stream. The product was obtained as shining dark-green crystals (yield: 190 mg; 84%).

Raman spectra of C_60_Pd_3_ were recorded using a Renishaw InVia Raman microscope equipped with a thermoelectrically TE-cooled charge-coupled detector. The excitation line length was 785 nm. The spectral resolution was better than 2 cm^–1^. The laser power at the sample was <1 mW. Raman spectra were recorded in the spectral range of 100–2000 cm^–1^ using a ×50 objective. To record Raman spectra as a function of pressure, a Merrill–Bessett diamond anvil was used. Hydrostatic pressure was transferred from the anvil piston to the sample using cesium iodide (CsI) as a medium and a stainless-steel seal. Ruby crystals were used for determining the value of the applied pressure. Type IIac diamonds (culet size: 0.7 mm) were embedded in BeCu anvil rings.

Absorption spectra were recorded using a Bruker Equinox 55 FT–IR spectrometer, coupled to a Hyperion 2000 microscope, in the KBr matrix (*c* = 1:50) in the mid-infrared range (650–4000 cm^–1^). The spectra were recorded with a spectral resolution of 2 cm^–1^. A tungsten lamp was used as the radiation source; liquid nitrogen-cooled mercury cadmium telluride detectors (500–7000 cm^–1^) were also used. KBr was used as a beam splitter. Infrared spectra were recorded as a function of pressure for C_60_, C_60_Pd_3_, and C_60_[η^2^-Pd­(PPh_3_)_2_]. A Merrill–Bessett high-pressure chamber was used for the experiments. Gasket Stainless was used as a seal between the diamonds. Potassium bromide (KBr) was used as a medium to transmit hydrostatic pressure from the anvil pistons to the sample (hydrostatic up to 2.1 GPa).^48,49^ Ruby crystals were used for determining the value of the pressure applied. Type IIac diamonds (culet size: 0.8 mm) embedded in BeCu anvil rings were used.

## Theoretical Calculations

Density functional theory (DFT) calculations were performed using the Gaussian09 package and the ωB97X-D^50,51^ hybrid density functional with a cep-121 basis set.^52^ For the theoretical C_60_ and C_60_Pd_3_ systems, geometry optimization was performed. For optimized geometries, normal mode calculations (theoretical IR and Raman spectra) were performed at the same level of theory. The results obtained for the calculated vibrational structure did not show any negative frequencies. This indicated that an energy minimum was obtained.

For the thermodynamic analysis, all initial geometries were prepared using the Avogadro 1.2.0 chemical editor.^53^ The Pd_13_ cluster was modeled as an icosahedron,^54^ while the initial structure of the Pd_12_ cluster was obtained by removing one Pd atom from the Pd_13_ cluster.

All DFT calculations were performed using the hybrid functionals ωB97X,^51^ ωB97X-D3,^55^ and ωB97X-V^56^ combined with the CRENBL effective core potential^57,58^ for carbon and palladium atoms (note: Gaussian software refers to CRENBL as CEP-121G) and the def2-TZVP basis set.^59^

Single-point energies at the ωB97X-D3/def2-TZVP and ωB97X-V/def2-TZVP levels of theory were calculated using the Orca 6.0.1 software.^60^ These calculations were performed on geometries previously optimized at the ωB97X/CRENBL level implemented in the TeraChem 1.93 software.^61^ Frequency calculations were also obtained at this level of theory and then scaled by a factor of 0.961 according to Scott and Radom.^62^ Low-frequency modes below 100 cm^–1^, which are common in Pd and Pd–C_60_ clusters, were increased to 100 cm^–1^ by using the quasi-harmonic correction method of Ribeiro et al.^63^ and Grimme^64^ to avoid overestimation of entropic contributions. These corrections improved the accuracy of thermodynamic properties by accounting for the anharmonic and fluxional motions characteristic of metal-containing systems.

The Gibbs free energy (*G*) of each system was obtained according to the equation

1 $$G=E_{\mathrm{elSP}}+\Delta E_{\mathrm{ZPE}}+\Delta H_{\mathrm{thermal}}-T\Delta S$$

where *E*_elSP_ denotes the electronic energy from the single-point calculation, while Δ*E*_ZPE_, *H*_thermal_, and *T*Δ*S* refer to the zero-point, enthalpic, and entropic corrections, respectively. Molecular volumes were calculated using the Monte Carlo algorithm implemented in the Multiwfn code.^65^

## Results and Discussion

### Raman and FTIR Spectra of C_60_Pd_3_ under Normal Pressure

To determine the effect of pressure on changes in C_60_Pd_3_, the pressure-dependent Raman and IR spectra were recorded at normal temperature. Herein, Raman spectra of the studied material exhibit a high degree of similarity to those of (C_60_)*_n_* obtained under high-pressure and -temperature conditions.^45^ Depending on the molar ratio of the precursors in the polymerization solution, C_60_Pd_*x*_ (*x* = 1–3) forms 1D, 2D, and 3D structures. The bands at 1447 and 1418 cm^–1^ indicate that each fullerene cage is coordinated by six Pd atoms to form a 3D C_60_Pd_3_ polymeric structure.

Remarkable peaks appear below 250 cm^–1^.^66^ This region exhibits peaks because of the intercage vibrations in different polymeric C_60_ materials such as photopolymers, polymeric C_60_ oxide phases, and C_60_ polymers formed under high-pressure and -temperature conditions.^37,66^ The intercage vibrations of C_60_ exhibit low intensity.^37,66^ C_60_Pd_3_ coordination polymers exhibit a substantially weak signal at 173 cm^–1^ characteristic of C_60_Pt_2_ and C_60_Pd_3_.^66^ Another typical feature of the transitional metal fullerides is the appearance of additional peaks in the region from 250 cm^–1^ to 800 cm^–1^.^66^ Several peaks are derived from original C_60_ modes (265, 288, 425, 486, 710, and 782 cm^–1^; Figure S2) because of the low symmetry of the polymeric phases. A splitting of C_60_ modes in this region appears in other fulleride materials.^66^

The important range of Raman spectra for metal–fullerene polymer is from 1400 cm^–1^ to 1600 cm^–1^, with the appearance of highly intense peaks (Figure 1 and Table 1). The peak at 1468 cm^–1^ is assigned to the characteristic A_g_(2) mode for C_60_ (Table 1), which is sensitive to chemical modifications.^66^ This peak for C_60_Pd_3_ downshifts to 1452 cm^–1^. For η^2^ fullerene complexes of Pt(0) and Pd(0), the downshift of the A_g_(2) mode is approximately 7–10 cm^–1^, while downshift is 5 cm^–1^ for C_60_Pd_2_ and C_60_Pt_2_.^66^

**Figure 1:**
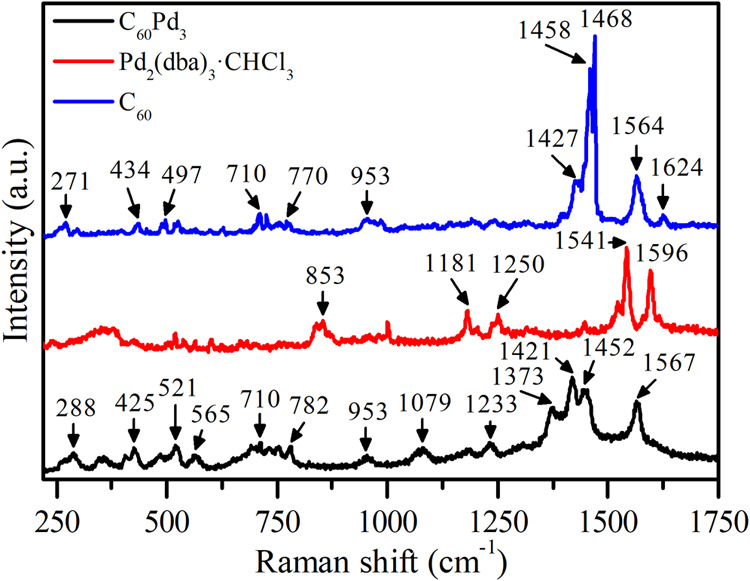
Raman spectra of C_60_Pd_3_ (black line), Pd_2_(dba)_3_·CHCl_3_ (red line), and C_60_ (blue line).

**Table 1:** Characteristic Raman and FTIR modes of C_60_Pd_3_ and C_60_ ^a^

	ν_Raman_ C_60_Pd_3_	ν_IR_ C_60_Pd_3_	ν_Raman_ C_60_	ν_IR_ C_60_	assignment	literature
1		1704 m		1733 w	ν(C=C) in C	this study
2		1615 m			ν(C=C) in C	this study
3	1567 s	1566 vw	1564 m		ν(C=C) in C	this study
4	1452 s	1448 m	1468 vs		polymer with metal intermolecular connections; A_g_(2) of C_60_	^66^
5	1421 s	1422 w	1407 w	1429 w	rhombohedral polymer; A_g_(2)	^45,67^
6	1373 m	1363 m	not observed		metal–C_60_ interaction	^45^
7	1233 w	1224 w	not observed	1262 s	probably metal–C_60_ interaction	this study
8		1156 w		1182 m		this study
9	1079 m	1085 vw	not observed	1094 s	metal–C_60_ interaction	^45^
10	953 m		953 w	1029 m	C_60_	this study
11	782 m	774 vw	770 w	806 m	C_60_	this study
12	710 m	698 m	710 w		C_60_	this study
13	565 m	559 m	not observed	576 w	probably lower symmetry in fulleride phases	^45,66^
14	521 m	519 vs	not observed	526 m	probably lower symmetry in fulleride phases	^45,66^
15	486 m	487 m	497 w		C_60_; Ag(2)	^45^
16	425 m	425 m	434 w		C_60_	this study
17	288 w		271 w		C_60_	this study
18	265 w		267 w		C_60_; Hg(1)	^45^
19	173 w				intercage vibrations of polymeric structure	^66^

a Legend: vs = very strong, s = strong, m = medium, w = weak, and vw = very weak.

The C_60_ band at 1407 cm^–1^ is assigned to the rhombohedral (C_60_)*_n_* homopolymer. The peak upshift to 1421 cm^–1^ for C_60_Pd_3_, indicating a change in the C_60_ symmetry. Additional peaks at 1373 and 1079 cm^–1^ appear in the Raman spectra of C_60_Pd_3_, which are absent for C_60_. The peaks at ∼1371 and 1076 cm^–1^ are also present in the spectra of other transition metal fullerides containing Nb, Ti, and Fe,^45,66^ corresponding to metal–C_60_ interactions.

Calculation was performed to examine the internal dynamic of the C_60_Pd_3_ system and was optimized using DFT/ωB97X-D/cep-121. FTIR and Raman spectra were calculated theoretically (Figure 2). Different colors are used to mark selected bands with their assignment. Selected modes are presented in color frames: gray (165 cm^–1^), blue (494 cm^–1^), black (1099 cm^–1^), and red (1490 cm^–1^). The other modes marked with different colors are presented in Figure S3. Characteristic modes for deformation of δ­(C–Pd–C) appear at 165 cm^–1^ (Figure 2a). Bands at 750, 1099, 1242, and 1431 cm^–1^ are related to the C–C bond of fullerene coordinated to Pd (Figures 2 and S3). The calculated results prove that the bands in the experimental Raman spectra of C_60_Pd_3_ appear at 1233 cm^–1^ because of the bonding of fullerene to Pd. The bands at 269, 494, 559, 1490, and 1657 cm^–1^ are characteristic of C_60_ (Figures 2 and S3).

**Figure 2:**
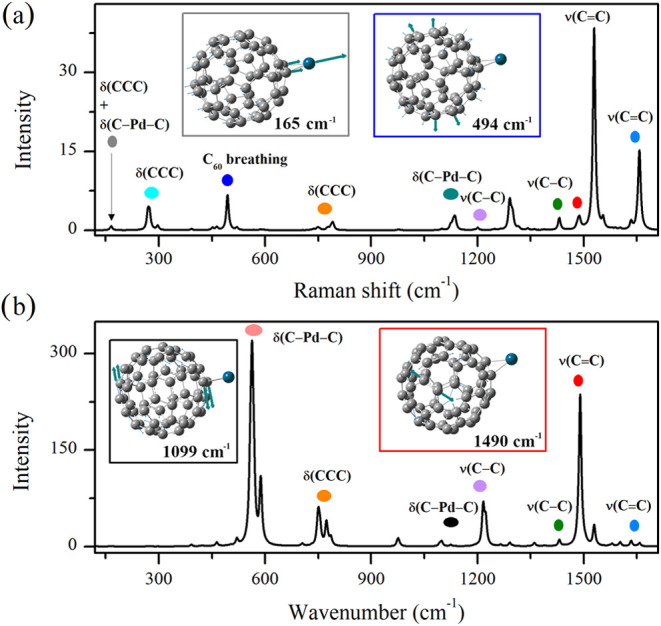
Calculated DFT/ωB97X-D/cep-121 for (a) Raman and (b) FTIR spectra of C_60_Pd_3_. Molecule–atom system (used in the calculation) with selected modes are presented in color frames: gray (165 cm^–1^), blue (494 cm^–1^), black (1099 cm^–1^), and red (1490 cm^–1^).

### Analysis of Raman and FTIR Spectra of C_60_Pd_3_ as a Function of Pressure

To investigate structural changes of C_60_Pd_3_ caused by increased pressure, Raman spectra were recorded (Figure 3). In the gray background, the diamond peak position is presented. Significant changes appear from 1000 cm^–1^ to 1650 cm^–1^ (Figure 3b). The intensity of bands at 1079, 1233, and 1373 cm^–1^ related to metal–C_60_ interaction decreases when the pressure increases. Under pressure of >4.75 GPa, these bands are not present in Raman spectra. Bands at 1421, 1452, and 1567 cm^–1^ shift to a higher wavenumber when the pressure increases (Figure 4a). The band at 1421 cm^–1^ shifts to 1466 cm^–1^ (∼45 cm^–1^ shift for changes in pressure from atmospheric pressure to 9.16 GPa), 1452 cm^–1^ to 1497 cm^–1^, and 1567 cm^–1^ to 1601 cm^–1^. The shift to a higher wavenumber indicates shortening of the C=C bonds in C_60_. Furthermore, shifting toward a higher wavenumber indicates increased C_60_ symmetry in the studied complex (Table 1). A shift of the band at 1421 cm^–1^ toward a higher wavenumber indicates a decrease in C_60_ and Pd energy bonding.^66^ Increasing the pressure from atmospheric to ∼4 GPa causes a shift in the band from 1452 cm^–1^ to 1468 cm^–1^. This band is characteristic of fullerene C_60_ and associated with Ag(2) vibrations (see Table 1). Burger et al. showed that this shift toward higher wavenumbers is associated with the formation of fullerene C_60_ dimers and trimers.^68^ Consequently, the shift of this band toward higher wavenumbers suggests the breakdown of bonds between C_60_ and Pd within the pressure range of 4 to ∼4.75 GPa.

**Figure 3:**
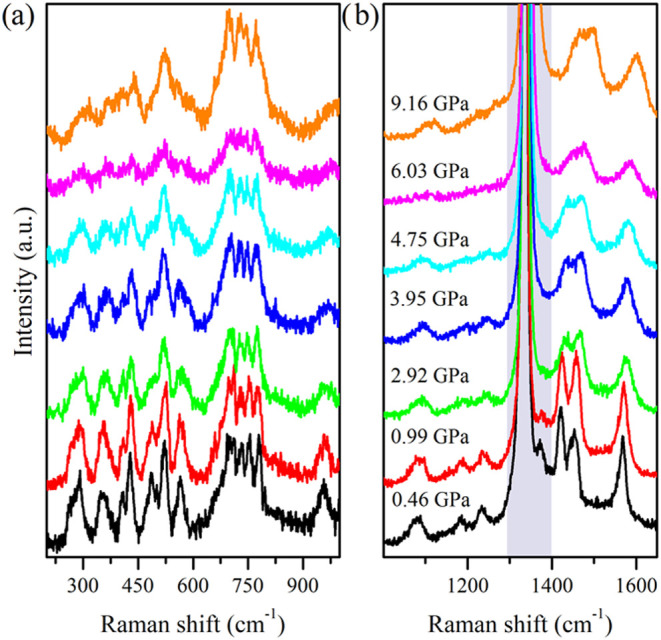
Pressure evolution of C_60_Pd_3_ Raman spectra in spectral ranges: (a) 200–1000 and (b) 1000–1650 cm^–1^. The diamond peak position is presented in the gray background.

**Figure 4:**
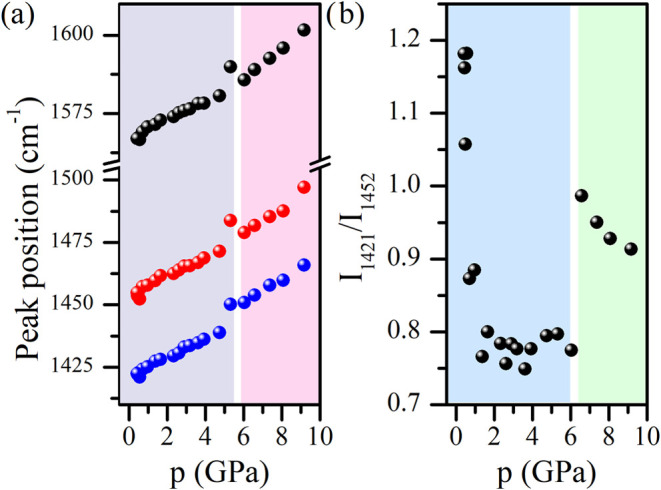
(a) Pressure evolution of 1567, 1452, and 1421 cm^–1^ Raman peak positions of C_60_Pd_3_. (b) Ratio of 1421 and 1452 cm^–1^ Raman band intensity.

The change in intensity of 1421, 1452, and 1567 cm^–1^ bands was analyzed to understand the structural changes of C_60_Pd_3_ under increasing pressure. At pressure lower than 0.5 GPa, the 1421 cm^–1^ band exhibits higher intensity than the 1452 cm^–1^ band (Figure 4b). The ratio of intensity of these bands (*I*_1421_/*I*_1452_) is equal to ca. 1.18. The relation between the intensity of both bands is reversed above 0.5 GPa, with a higher intensity of the 1452 cm^–1^ band than that of the 1421 cm^–1^ band. The *I*_1421_/*I*_1452_ ratio approaching value of ca. 0.77. At these pressures, the C_60_Pd_3_ polymer brakes down to C_60_Pd_2_ in which each fullerene moiety is coordinated to four palladium atoms to form 2-dimensional polymeric network.^66^ Another significant structural changes are observed at pressure of about 6 GPa. At that pressure, step change in the relation between the intensity of 1421 and 1452 cm^–1^ bands appears (Figure 4b). Then, as the pressure changes from 6 to 9.15 GPa, the *I*_1421_/*I*_1452_ ratio decreases from 0.99 to 0.92, respectively. Such behavior suggests that palladium linkers are released from the polymeric phase and C_60_ homopolymer is formed. The formation of C_60_ homopolymeric phase at 5 GPa at high temperatures was also reported by Iwasa and co-workers.^69^ Described structural changes are schematically shown in Scheme 2.

**Scheme 2:**
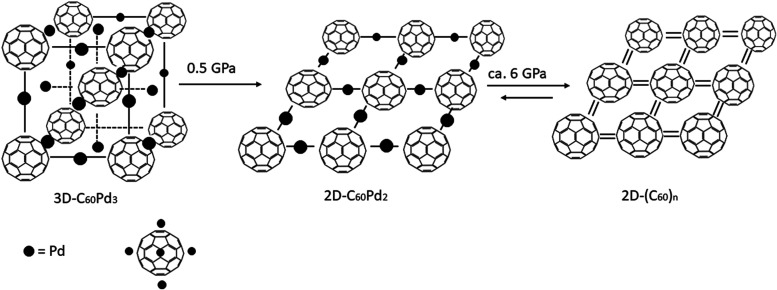
Schematic Illustration of C_60_Pd_3_ Coordination Polymer Structural Changes upon Increasing Pressure

Analysis of the half widths of 1421, 1452, and 1567 cm^–1^ bands as a function of pressure shows three pressure ranges, exhibiting trend changes (Figure S4). From atmospheric pressure to ∼4 GPa, the half widths of the bands at 1421, 1452, and 1567 cm^–1^ change by ∼17, 6, and 11 cm^–1^, respectively. In the range of 6–9 GPa for the 1421 and 1452 cm^–1^ bands, the half width changes by ∼3.5 and 1.5 cm^–1^, respectively. The step change of the 1567 cm^–1^ band appears at ∼6 GPa. The evolution trend also changes, and a decrease in the half width of the 1567 cm^–1^ band appears between 6 and 9 GPa (Figure S4c). Above 6 GPa, 521 and 486 cm^–1^ bands disappear, and a new band appears at 516 cm^–1^ (Figure S5).

The effect of the pressure on the FTIR spectra recoded for C_60_Pd_3_ are shown in Figure S6. The most significant changes in the spectroscopic properties of studied coordination polymer are observed in the wavenumber range of 1000–1500 cm^–1^ (Figure 5a). For the pressure higher than ca. 0.5 GPa, an additional highly intense band appears at 1389 cm^–1^, which is probably related to the C–C bond stretching vibration of carbon atoms linked to Pd atoms in two-dimensional C_60_Pd_2_ polymeric network. The band shifts toward a higher wavenumber with increasing pressure (from 1389 cm^–1^ to 1397 cm^–1^), indicating that the distance between carbon atoms bonded to Pd and between fullerene cages in the polymeric phase becomes shorter. At ca. 4 GPa, a strong band at ca. 1400 cm^–1^ disappears, with the appearance of two broad bands of low intensity between 1300 and 1550 cm^–1^. These broad bands indicate formation of fullerene phase in which fullerene moieties are bounded through double bond to form polymeric phase.

**Figure 5:**
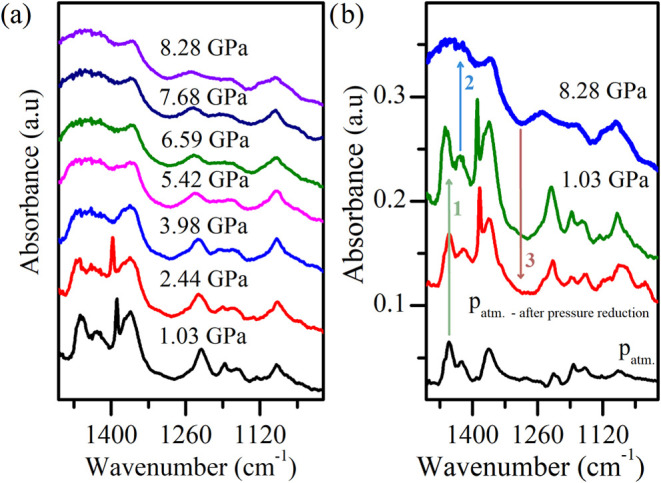
FTIR spectra of (a) C_60_Pd_3_ under increasing pressure and (b) at atmospheric pressure before increasing pressure (black line described by *p*_atm_), at 1.03 and 8.28 GPa during increasing preassure, and at atmospheric pressure after pressure reduction (red line). Arrows indicate sequence of pressure changes during measurements.

To determine whether the observed transformation is reversible, a comparative analysis was performed on Raman and FTIR spectra recorded after reducing the pressure from high value of 8.28 GPa to atmospheric level (Figure 5b). A sharp band near 1389 cm^–1^ does not appear for pristine C_60_Pd_3_ not subjected to high pressure. However, when the pressure is reduced from 8.28 GPa to atmospheric pressure, the band at ca. 1389 cm^–1^ appears in the FTIR spectrum of the investigated sample. This sharp band does not appear for starting material, pristine C_60_Pd_3_, not subjected to high pressure. Such behavior suggests that removing the high pressure subjected sample from the pressure chamber and placing it under normal atmospheric pressure introduce the formation of C_60_Pd_2_ polymeric network. However, under these conditions, the formation of the initial polymer phase C_60_Pd_3_ is not observed.

The results of Raman studies provide more insight into the behavior of the tested polymer material under conditions of pressure reduction from 9.15 GPa to atmospheric pressure (Figure S7). Observation of the material under a microscope shows the formation of two phases when the pressure is reduced from 9.16 GPa to atmospheric pressure. One of them shows the structure of a fullerene coordination polymer with carbon networks bonded via palladium atoms. Bands at 1421 and 1452 cm^–1^ are present in the Raman spectrum. They are slightly shifted toward higher wavnumbers by about 3 cm^–1^. The second phase exhibits Raman spectroscopy behavior typical for homofullerene polymer.

The results presented above qualitatively correlate well with the previously described changes in the intensity of Raman peaks (Figure 4). However, they indicate that changes leading to the removal of palladium linkers from the coordination polymer phase may occur at a slightly lower pressure than suggested by the experimental results presented in Figure 4.

For comparison, the influence of pressure on the IR spectra of pristine C_60_ was investigated (Figure S8). The red and orange spectra were recorded for decreasing pressure after reaching 8.99 GPa. As the pressure increases, the 806 and 1182 cm^–1^ bands shift toward higher and lower wavenumbers, respectively. The 1262 cm^–1^ band begins to shift toward a lower wavenumber at 3.67 GPa. Additionally, the 1094 cm^–1^ band shifts toward a higher wavenumber when the pressure is increased to 1.84 GPa. Further increase in pressure does not cause any changes. These bands broaden, which might indicate an increase in the interaction energy between successive fullerenes. The spectrum of C_60_ reverts to its original form when the pressure is reduced from 8.99 to 0.35 GPa, indicating a reversible change of pristine C_60_ after being subjected to pressure changes. Increasing the pressure increases the bond strength between fullerenes. However, it is not sufficient to form a C_60_=C_60_ homopolymer phase.

### Analysis of FTIR Spectra of a C_60_[η^2^-Pd­(PPh_3_)_2_] Complex as a Function of Pressure

The pressure effect on C_60_[η^2^-Pd­(PPh_3_)_2_] was analyzed to determine whether a homopolymeric (C_60_)*_n_* phase could be formed under elevated pressure. Figure S9 shows the FTIR spectrum of C_60_[η^2^-Pd­(PPh_3_)_2_], marking the most intense bands characteristic of C_60_: 1262, 1186, 1027, 806, 741, and 696 cm^–1^ (Table 1). Bands at 1435, 1358, 1097, and 521 cm^–1^ indicating C_60_ connected to the metal center (Table 1),^42^ and at 1483 and 1463 cm^–1^ associated with the PPh–Pd bonds are also present.

Figure S10 shows the pressure evolution of C_60_[η^2^-Pd­(PPh_3_)_2_]. As the pressure increases from atmospheric pressure to 8.32 GPa, the intensity of all bands decreases drastically. In the range of 5.57–8.32 GPa, no bands appear in the 1300–900 cm^–1^ region, which are characteristic of fullerene–Pd bonds. The 1483 and 1463 cm^–1^ bands at 3.57 GPa convert into a single band and drastically decrease in intensity as the pressure is increased. As the pressure is reduced, fewer bands are observed in the FTIR spectrum of C_60_[η^2^-Pd­(PPh_3_)_2_]. Bands at 1435, 694, and 522 cm^–1^ represent the palladium-linked polymer but with reduced intensity. The observed changes indicate the presence of an amorphous phase in the pressure range of 5.57–8.32 GPa. When the pressure is reduced to atmospheric pressure, two phases are identified in the spectrum: the amorphous phase (the dominant phase) and C_60_[η^2^-Pd­(PPh_3_)_2_].

### Thermodynamic Analysis

In the process of the (C_60_)*_n_* homopolymeric phase formation, two main reactions are involved [eqs 2 and 3].

2 $$\left(R1\right),2n\;C_{60}\to \text{−}{\left(C_{60}=C_{60}\right)}_{n}\text{−}$$

3 $$\left(R2\right),\text{−}{\left(\mathrm{Pd}\text{−}C_{60}\right)}_{n}-+Pd\left(\mathrm{bulk}\right)\to \text{−}{\left(C_{60}=C_{60}\right)}_{n}-+\left[n\mathrm{Pd}+\mathrm{Pd}\left(\mathrm{bulk}\right)\right]$$

Based on these processes, we considered the following reactions in our theoretical modeling.

4 $$\left(r1\right),2C_{60}\to C_{60}=C_{60}$$

5 $$\left(r2\right),C_{60}\text{−}\mathrm{Pd}\text{−}C_{60}+\mathrm{Pd}\left(\mathrm{bulk}\right)\to C_{60}=C_{60}+\left[\mathrm{Pd}+\mathrm{Pd}\left(\mathrm{bulk}\right)\right]$$

6 $$\left(r3\right),\mathrm{Pd}\text{−}C_{60}\text{−}\mathrm{Pd}\text{−}C_{60}+\mathrm{Pd}\left(\mathrm{bulk}\right)\to C_{60}=C_{60}+\left[2\mathrm{Pd}+\mathrm{Pd}\left(\mathrm{bulk}\right)\right]$$

7 $$\left(r4\right),\mathrm{Pd}\text{−}C_{60}+C_{60}+\mathrm{Pd}\left(\mathrm{bulk}\right)\to C_{60}=C_{60}+\left[\mathrm{Pd}+\mathrm{Pd}\left(\mathrm{bulk}\right)\right]$$

The first reaction (r1) corresponds to the process of the homopolymer synthesis from pristine C_60_ molecules (R1). The other reactions (r2, r3, and r4) refer to the formation of the homopolymer from (PdC_60_)*_n_* in the presence of palladium (R2). The calculated values of the enthalpy (Δ*H*), entropy (Δ*S*), Gibbs free energy (Δ*G*), and molecular volume (V) for substrates and products are summarized in Table S1.

The Gibbs free energy (Δ*G*_rn_) of reactions r1–r4 was calculated using eqs 8–11.

8 $$\Delta G_{r1}=G\left(C_{60}=C_{60}\right)-2G\left(C_{60}\right)$$

9 $$\Delta G_{r2}=G\left(C_{60}=C_{60}\right)-G\left(C_{60}\text{−}\mathrm{Pd}\text{−}C_{60}\right)+\Delta G_{\mathrm{coh}}$$

10 $$\Delta G_{r3}=G\left(C_{60}=C_{60}\right)-G\left(\mathrm{Pd}\text{−}C_{60}\text{−}\mathrm{Pd}\text{−}C_{60}\right)+2\Delta G_{\mathrm{coh}}$$

11 $$\Delta G_{r4}=G\left(C_{60}=C_{60}\right)-\left[G\left(\mathrm{Pd}\text{−}C_{60}\right)+G\left(C_{60}\right)\right]+\Delta G_{\mathrm{coh}}$$

The cohesion free energy (Δ*G*_coh_) was estimated as the mean difference between the Gibbs free energy (*G*) of Pd binding to and removing from the icosahedral Pd_13_ cluster

12 $$\Delta G_{\mathrm{coh}}=\left[G\left({\mathrm{Pd}}_{14}\right)-G\left({\mathrm{Pd}}_{12}\right)\right]/2$$

Next, we included the pressure dependence on Δ*G* (Δ*G*_p_ = *p*·Δ*V*) via *V* change (Δ*V*) during the polymerization reaction. Thus, we analyzed the pressure contribution to Δ*G*_p_ for all considered reactions. The molecular volumes (*V* in Å^3^) were taken from the structural data (Tables S1 and S2) and converted into molar *V* (cm^3^ mol^–1^). The calculations were performed at the temperature (*T*) of 298 K and pressures up to 30 GPa to reproduce the experimental conditions. The change of reaction volume (Δ*V*) was calculated as Δ*V* (Å^3^) = ∑*V*_products_ – ∑*V*_reactants_. The minor volume change accompanying the transition of Pd atom(s) into the bulk phase was neglected. The conversion into molar volume was performed using Δ*V* (cm^3^ mol^–1^) = Δ*V* (Å^3^) × 0.602214, where 0.602214 cm^3^ mol^–1^ corresponds to 1 Å^3^ per molecule multiplied by Avogadro’s number.

Our results demonstrate that pressure has a strong influence on fullerene dimerization in the presence of Pd clusters (Figures 6 and S11). The stabilizing effect of pressure on this process is related to the reduction in Δ*V*. The pressure dependence of ΔG indicates that all four reactions become highly favorable as pressure increases. However, this dependence is stronger for the reactions involving the C_60_–Pd binding motif. Consequently, we can extrapolate our findings to the formation of (C_60_)*_n_* and confirm that it forms from C_60_Pd_3_ at substantially lower pressure than from amorphous fullerene, as observed experimentally.

**Figure 6:**
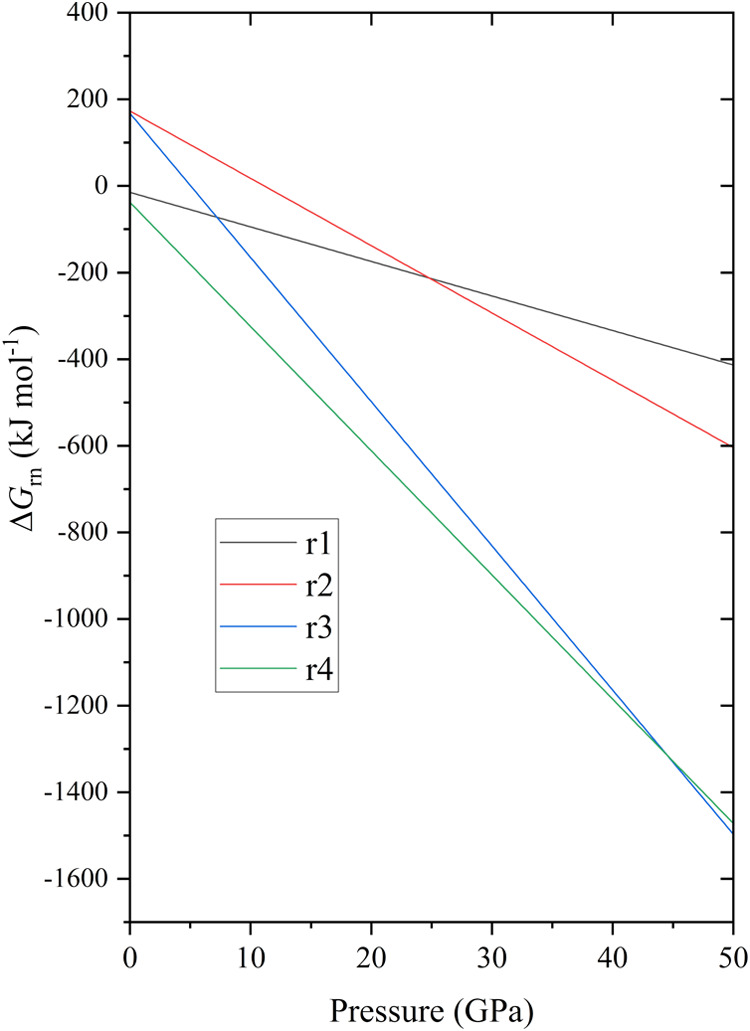
Pressure dependence of Δ*G*_*rn*_(p) for model reactions (r1–r4) calculated at the ωB97X-D3/def2-TZVP//ωB97X/CRENBL level of theory.

### Explanation of Phase Transitions in C_60_Pd_3_ under High Pressure

The sample was analyzed spectroscopically to clarify the changes that occurred in C_60_Pd_3_ under high pressure. It comprises two distinct phases: m1 and m2. These phases are visible under a microscope with an objective ×50 (photo in the upper left corner of Figure 7). Raman spectra were recorded for sites m1 (Spectrum 1; Figure 7a) and m2 (Spectrum 2; Figure 7a) at atmospheric pressure after the pressure was reduced from 9.16 GPa. Spectrum 1 is similar to that of C_60_Pd_3_ (Figure 1), indicating the presence of C_60_–Pd. However, spectrum 2 is entirely different, which is identical to the Raman spectrum of C_60_ in the amorphous phase obtained for C_60_/C_70_ subjected to a pressure of 36 GPa.^70^ A similar spectrum appears for the homopolymer C_60_=C_60_ (spectrum 3; Figure 7a). This analysis shows that the sample at m2 (Figure 7a) exhibits an amorphous phase of C_60_ in which a double bond links C_60_ moieties to form the disordered homopolymer phase of C_60_.

**Figure 7:**
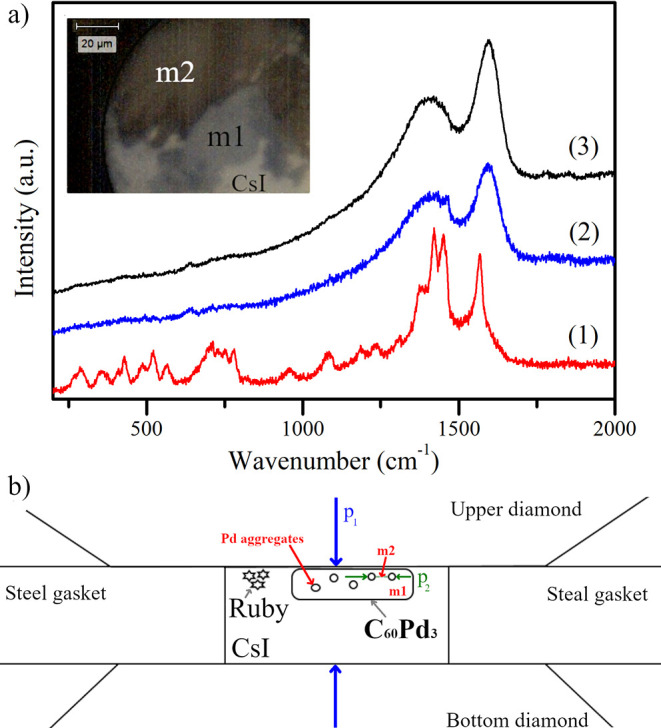
(a) Raman spectra of m1 (1), m2 (2), and C_60_ homopolymer (3). m1 and m2 are marked in the picture in the left corner and were recorded at atmospheric pressure after the pressure was reduced from 9.16 GPa. (b) Schematic illustration of pressure-induced changes in C_60_Pd_3_.

XRD analysis reveals that decomposition of the polymeric phase of C_60_Pd_3_ leads to the aggregation of Pd nanoparticles into large grains.^45^ However, Pd nanoparticles are formed at slightly low temperature.^45^ The Raman and FTIR spectra show that at pressures of >4 GPa, an amorphous phase of C_60_ appears in a certain volume of the studied sample, similar to C_60_/C_70_ at 36 GPa.^70^ This behavior is related to the aggregation of Pd nanoparticles into larger grains, similar to the behavior reported by Talyzin et al. for C_60_Pd_3_ heated to high temperatures.^45^ Analysis of the C_60_ phase diagram reveals that an amorphous C_60_ phase can be obtained at 20 °C above a pressure of 15 GPa.^71^ In our experiments, the (C_60_)*_n_* homopolymeric phase is formed at a considerably low pressure of 4 GPa. The appearance of the homopolymer phase at ∼4 GPa is attributed to the reduced energy of fullerene after Pd atoms are attached to it. However, analysis of C_60_[η^2^-Pd­(PPh_3_)_2_] shows that attaching the η^2^-Pd­(PPh_3_)_2_ group to fullerene does not reduce its energy sufficiently to allow homopolymer formation. This possibility is probably also owing to the high local pressure between Pd aggregates formed during polymeric phase decomposition.

Pd aggregates increase the local chemical pressure (Figure 7b). Applying greater pressure to the sample under investigation by using two diamonds (p1; Figure 7b) induces Pd aggregates to move closer, which subsequently causes a localized increase in pressure between them (p2; Figure 7b). This mechanism is similar to using additional diamond crystals in the diamond chamber, which form the environment closest to the studied sample.^72,73^

The above interpretation of experimental results is confirmed through a theoretical calculation of the fullerene cage dimerization process in the presence of Pd nanoclusters. These calculations confirm the stabilizing effect of pressure on fullerene dimerization and the related Pd-assisted reactions, as a consequence of the negative reaction volume (Δ*V*). Thus, metal-assisted preorganization represents an efficient strategy for promoting polymerization under milder conditions.

## Conclusions

Herein, the pressure effect on the structural changes of C_60_Pd_3_ is described. The structural changes of the polymeric phase on increasing pressure were monitored via in situ FTIR and Raman spectroscopy. With increasing pressure, 3D body-centered cubic structure of fullerene moieties coordinated by six Pd atoms in an octahedral fashion was decomposed to form linear C_60_Pd polymer chains. At elevated pressure of ca. 4.5 GPa, the formation of an amorphous (C_60_)*_n_* homofullerene polymeric phase was observed. Results of FTIR and Raman spectroscopic studies obtained for C_60_Pd_3_ were compared with the results of analogous investigations performed using pristine fullerene C_60_ and C_60_[η^2^-Pd­(PPh_3_)_2_]. Placing C_60_Pd_3_ in a diamond chamber increased the local pressure owing to the ease of forming Pd aggregates. Under a highly increased local pressure, the (C_60_)*_n_* homopolymer was formed, which was not observed for pristine fullerene and C_60_[η^2^-Pd­(PPh_3_)_2_]. The additional theoretical calculations showed that pressure strongly influenced the bonding of fullerene moieties in the presence of Pd clusters, which was particularly significant in the case of C_60_ moieties linking by palladium atoms.

## Supplementary Material



## Notes

Pressure dependence of FTIR and Raman spectra of the C_60_Pd_3_, C_60_, and C_60_[η^2^-Pd­(PPh_3_)_2_]; pressure evolution of Raman half-width and peak positions; thermodynamic functions and pressure contributions to Gibbs free energy at 298 K; pressure dependence of Δ*G* at *T* = 298 K; XRD spectrum of as prepared C_60_Pd_3_ polymer (PDF)
