# Supplementary material for: In Situ Infrared and Raman Analysis of Structural Changes of (C60Pd3) n Coordination Polymer under Elevated Pressure
Source: J Phys Chem B. 2026 Apr 14;130(16):4473–82. doi: 10.1021/acs.jpcb.5c08143 (PMC13299011; doi:10.1021/acs.jpcb.5c08143)
Supplement: Supplementary file 1 [file jp5c08143_si_001.pdf]

Supporting Information: In Situ Infrared and Raman Analysis of Structural Changes of $(C_{60}Pd_3)_n$ Coordination Polymer under Elevated Pressure

Sylwia Zięba,[1]* Emilia Grądzka,[2] Adam Mizera,[1] Dariusz Pogocki,[3] Barbara Seroka,[2] Jakub Goclon,[2] Ryszard Łaźny,[2] Krzysztof Winkler[2]

[1]Institute of Molecular Physics, Polish Academy of Sciences, M. Smoluchowskiego 17, 60-179 Poznań, Poland

[2]Department of Chemistry, University of Bialystok, K. Ciołkowskiego 1K, 15-245 Bialystok, Poland

[3]Institute of Nuclear Chemistry and Technology, Dorodna 16, 03-195 Warsaw, Poland

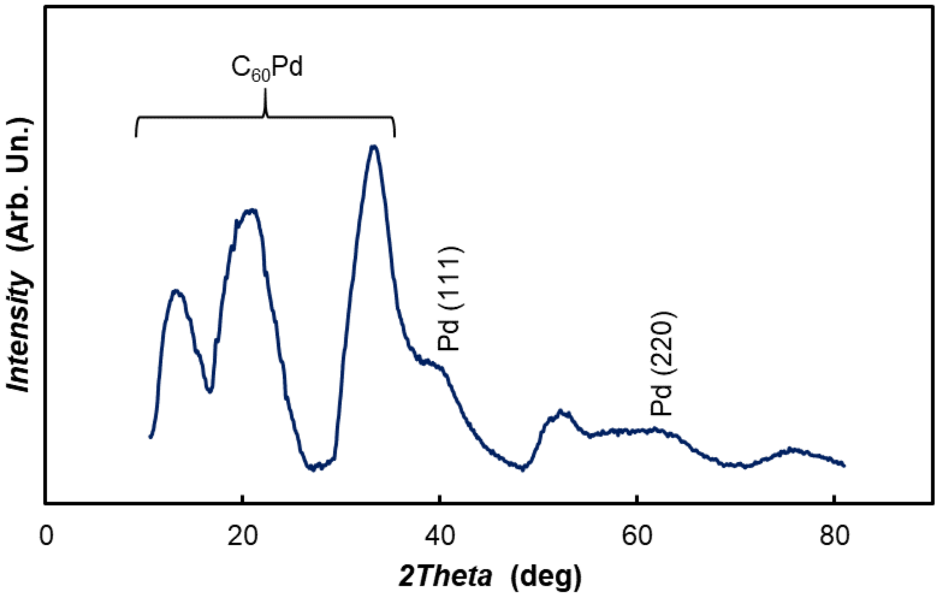

Figure S1. XRD pattern of as prepared $C_{60}Pd_3$ polymer.

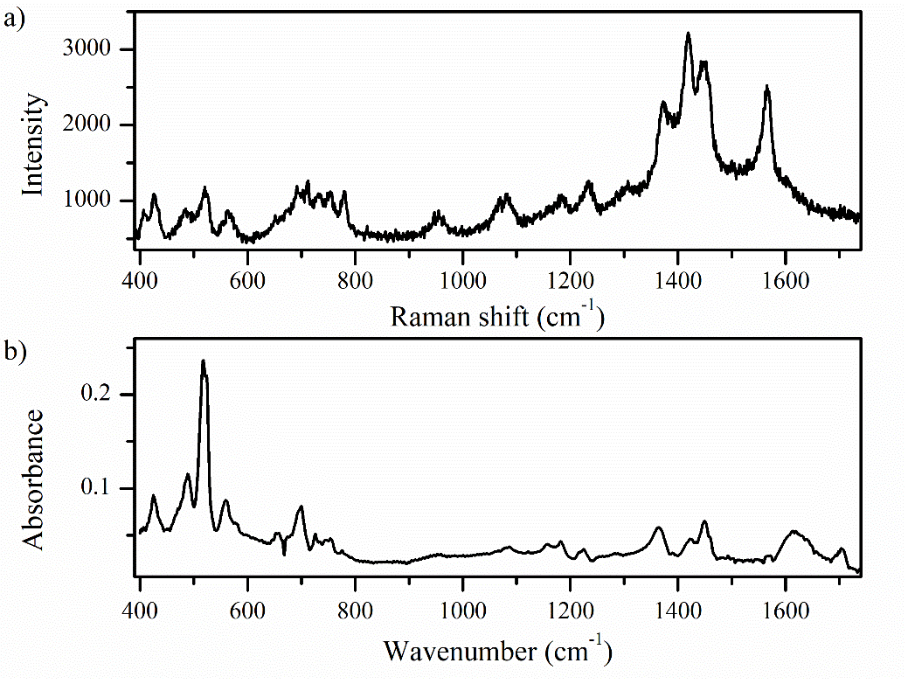

Figure S2. Raman spectrum (a) and FT-IR spectrum (b) of $C_{60}Pd_3$. FT-IR spectrum measured in KBr matrix (*c*=1:500).

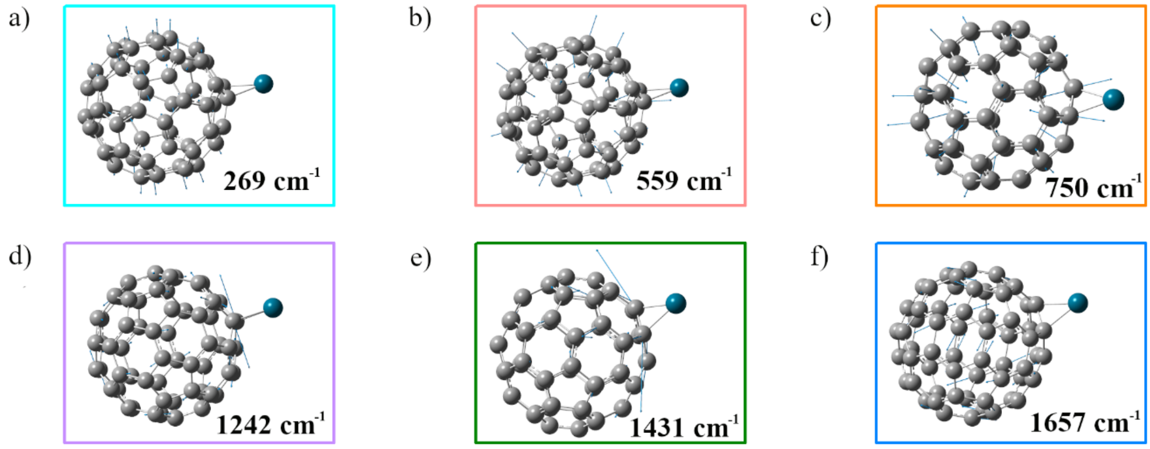

Figure S3. Selected modes of calculated DFT/ωB97XD/cep-121 molecule-atom system. The position of these modes in Raman and FT-IR spectra were presented in **Figure 2,** which are the same colors as the frames.

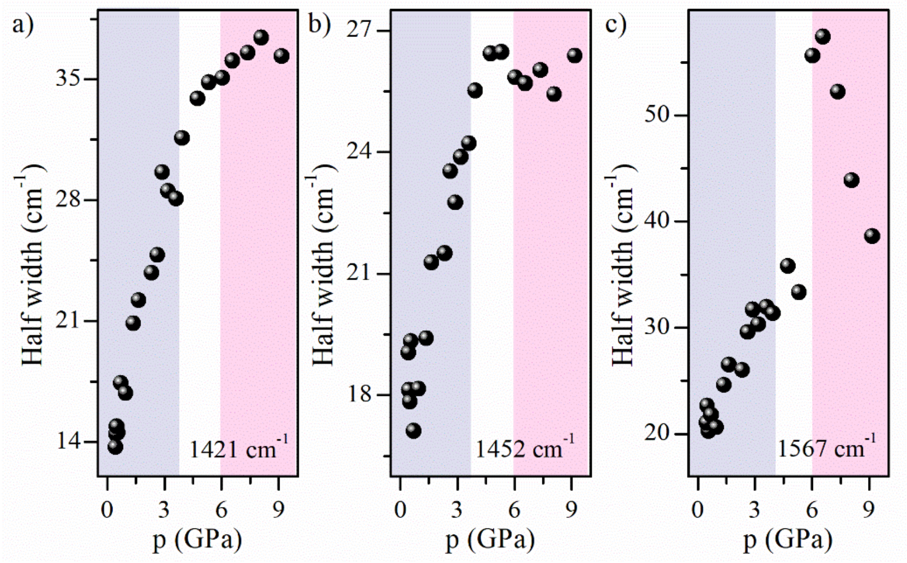

Figure S4. Pressure evolution of the half-width of the bands observed in Raman spectra for $C_{60}Pd_3$: 1421 (a), 1452 (b), and 1567 cm^{-1} (c).

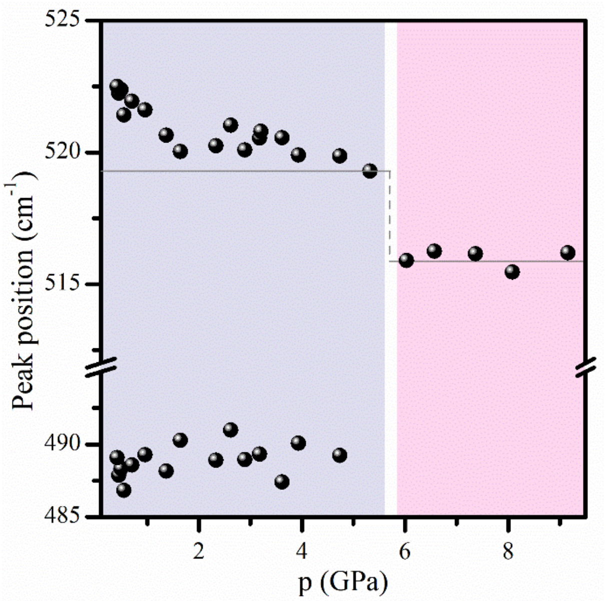

Figure S5. Pressure evolution of the peak positions at 521 and 486 cm^{-1} observed in Raman spectra for $C_{60}Pd_3$.

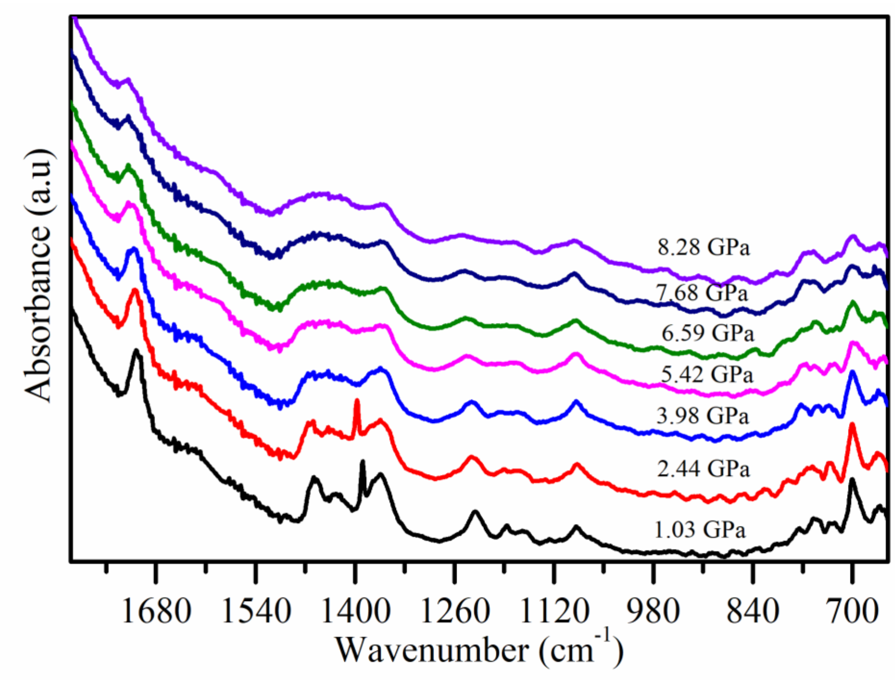

Figure S6. FT-IR spectra under pressure of $C_{60}Pd_3$ in KBr matrix (c=1:50).

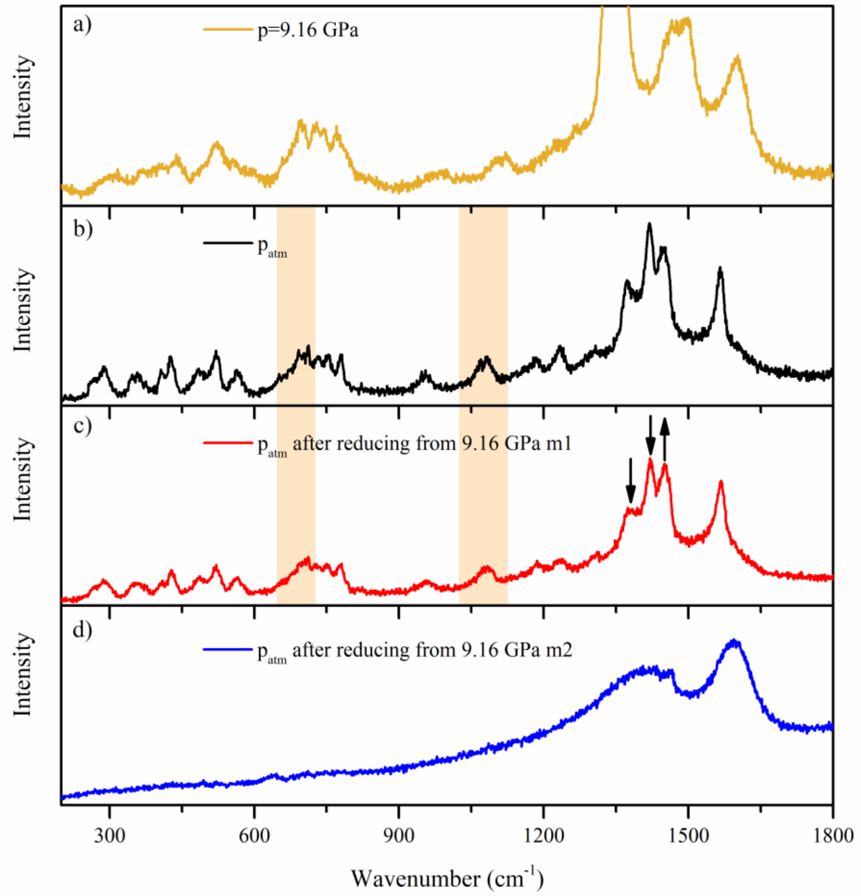

Figure S7. Raman spectra of $C_{60}Pd_3$ at 9.16 GPa (a), atmospheric pressure (b), atmospheric pressure after reducing pressure from 9.16 GPa for m1 (c) and m2 (d) places.

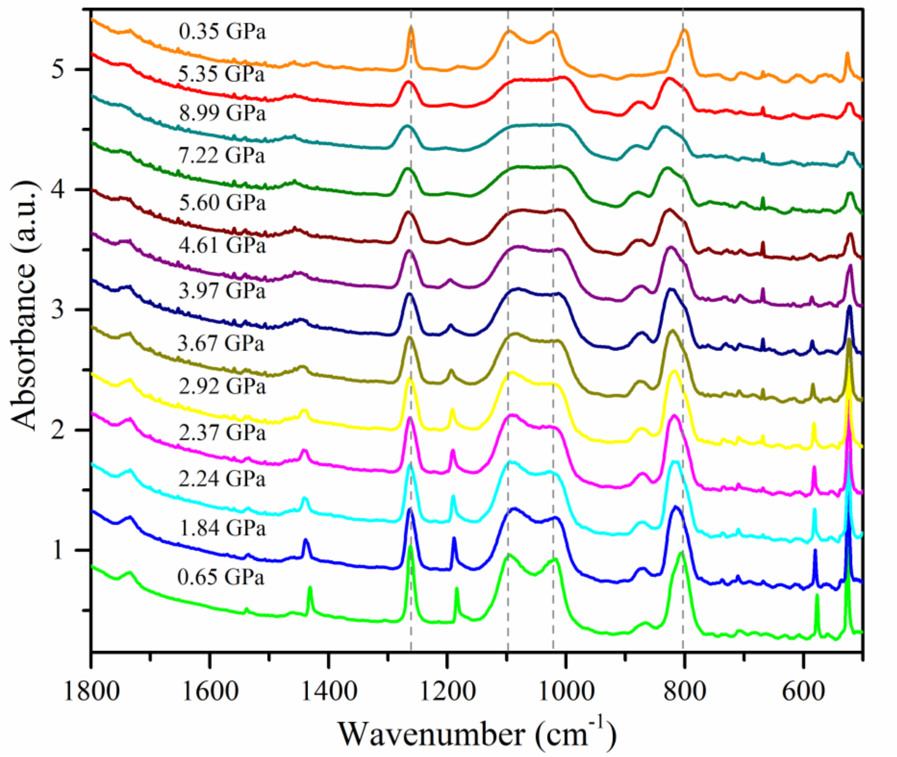

Figure S8. FT-IR spectra under pressure of C_{60} in KBr matrix (*c*=1:50). Spectra at 5.35 and 0.35 GPa are marked in red and orange, respectively for pressure decrease.

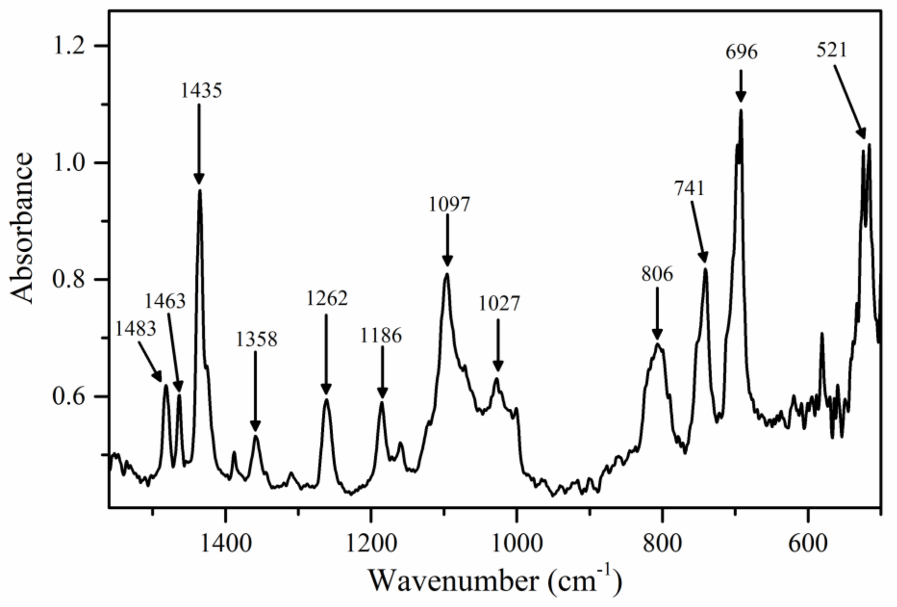

Figure S9. FT-IR spectrum of $C_{60}Pd(PPh_3)_2$ in KBr matrix (*c*=1:50).

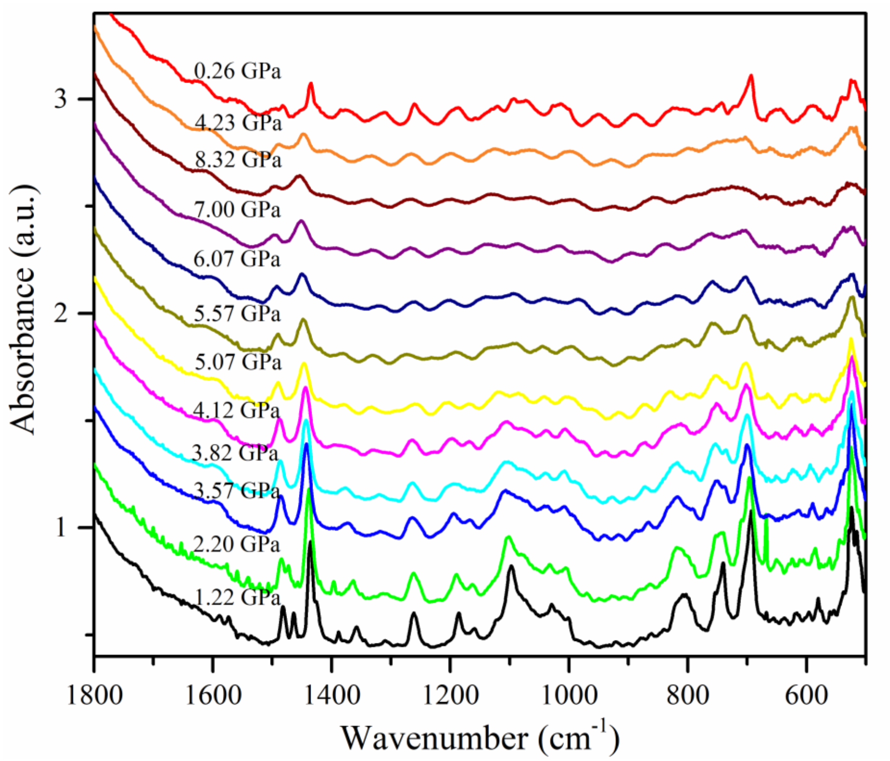

Figure S10. FT-IR spectrum under the pressure of $C_{60}Pd(PPh_3)_2$ in KBr matrix (*c*=1:50). Spectra at 4.23 and 0.26 GPa are marked in orange and red respectively for pressure decrease.

Thermodynamic analysis

Table S1. Molecular volumes (V), enthalpies (Δ*H*), entropies (Δ*S*) and Gibbs free energies (Δ*G*) at 298 K calculated at the ωB97X-D3/def2-TZVP//ωB97X/CRENBL level of theory.

	V ($Å^3$)	$\Delta H^{\varnothing}_{298}$ (Hartree)	$\Delta S^{\varnothing}_{298}$ (kJ mol^{-1} K^{-1})	$\Delta G^{\varnothing}_{298}$ (Hartree)
C_{60}	635.8	-2285.8656	0.076	-2285.8742
C_{60}=C_{60}	1258.4	-4571.7116	0.383	-4571.7539
Pd-C_{60}	670.2	-2413.8229	0.149	-2413.8398
C_{60}-Pd-C_{60}	1284.3	-4699.7338	0.467	-4699.7868
Pd-C_{60}-Pd-C_{60}	1313.7	-4827.6933	0.515	-4827.7518
Pd_{12}-C_{60}-Pd-C_{60}	1594.5	-6235.2399	0.906	-6235.3428
Pd_{12}	----	-1535.3739	0.063	-1535.3810
Pd_{13}	----	-1663.3463	0.080	-1663.3554
Pd_{14}	----	-1791.3008	0.125	-1791.3150

Table S2. Molecular volumes (V), enthalpies (Δ*H*), entropies (Δ*S*) and Gibbs free energies (Δ*G*) at 298 K calculated at the ωB97X-V/def2-TZVP//ωB97X/CRENBL level of theory.

	V ($Å^3$)	$\Delta H^{\varnothing}_{298}$ (Hartree)	$\Delta S^{\varnothing}_{298}$ (kJ mol^{-1} K^{-1})	$\Delta G^{\varnothing}_{298}$ (Hartree)
C_{60}	635.8	-2286.0132	0.076	-2286.0218
C_{60}=C_{60}	1258.4	-4572.0136	0.415	-4572.0607
Pd-C_{60}	670.2	-2413.9075	0.149	-2413.9244
C_{60}-Pd-C_{60}	1284.3	-4699.9660	0.467	-4700.0190
Pd-C_{60}-Pd-C_{60}	1313.7	-4827.8629	0.515	-4827.9214
Pd_{12}-C_{60}-Pd-C_{60}	1594.5	-6234.6887	0.906	-6234.7915
Pd_{12}	----	-1534.5810	0.063	-1534.5881
Pd_{13}	----	-1662.4888	0.080	-1662.4978
Pd_{14}	----	-1790.3737	0.125	-1790.3878

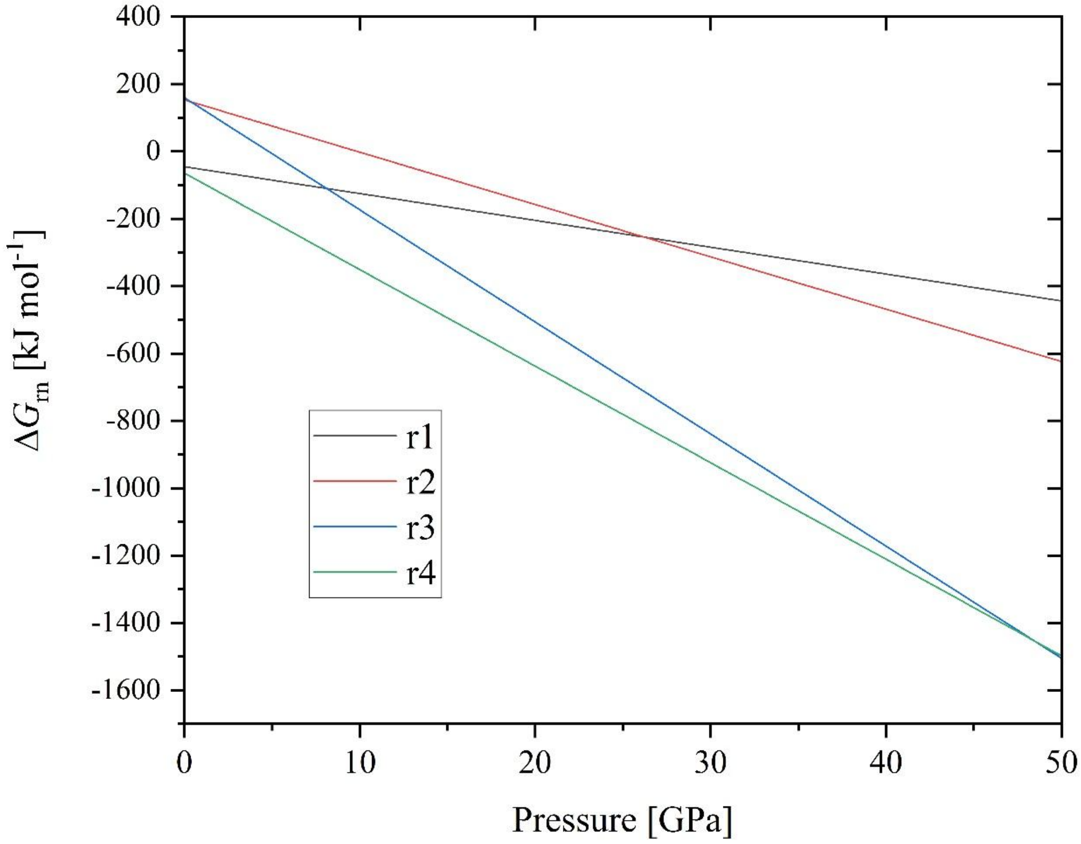

Figure S11. Pressure dependence of Gibbs free energy ΔG_{rn}(p) for model reactions (r1–r4) calculated at the ωB97X-V/def2-TZVP//ωB97X/CRENBL level of theory.
